# C3 glomerulopathies: dense deposit disease and C3 glomerulonephritis

**DOI:** 10.3389/fmed.2023.1289812

**Published:** 2023-11-24

**Authors:** Claudio Ponticelli, Marta Calatroni, Gabriella Moroni

**Affiliations:** ^1^Independent Researcher, Milan, Italy; ^2^IRCCS Humanitas Research Hospital, Milan, Italy; ^3^Department of Biomedical Sciences, Humanitas University, Milan, Italy

**Keywords:** C3 glomerulopathies, dense deposit disease, C3 glomerulonephritis, membra-noproliferative glomerulonephritis, alternative complement pathway

## Abstract

Dense deposit disease (DDD) and C3 glomerulonephritis (C3GN) are types of membranoproliferative glomerulonephritis classified as C3 glomerulopathies. These conditions are characterized by an increased number of intraglomerular cells and diffuse thickening of the glomerular capillary walls, along with the deposition of C3 and minimal or absent immunoglobulin deposits. The underlying cause of both DDD and C3Gn is an abnormal activation of the alternative complement pathway, which can result from acquired or genetic alteration. In acquired forms of DDD and C3GN, the dysregulation of the alternative pathway is commonly induced by the presence of C3 nephritic factors (C3NeFs), which are autoantibodies that stabilize C3 convertase. Both DDD and C3GN can affect individuals of any age, but DDD is primarily diagnosed in children, whereas C3GN tends to be diagnosed at a significantly higher age. The presenting features of these diseases are variable and may include proteinuria, hematuria, hypertension, or kidney failure. A common finding in these diseases is low serum C3 levels with normal serum C4 levels. Chronic deterioration of renal function is commonly observed in DDD and C3GN, often leading to end-stage renal disease (ESRD), especially in DDD. Kidney transplantation outcomes in patients with these conditions are characterized by histological recurrence, which may contribute to higher rates of allograft failure.

## Highlights

- C3 glomerulopathies include dense deposit disease (DDD) and C3 glomerulonephritis (C3GN).
- Both diseases result from acquired or genetic abnormal activation of the alternative complement pathway.
- DDD affected primarily children, while C3GN is more frequent in adult patients.
- Clinical presentation is variable, with proteinuria, hematuria, hypertension, or kidney failure. Persistent low C3 and normal C4.
- Main histological features are an increased number of intraglomerular cells and diffuse thickening of the glomerular capillary. C3 deposition with absent or scanty deposits of immunoglobulins.
- Both diseases lead to end-stage renal disease with high recurrence after transplant.

## Introduction

Membranoproliferative glomerulonephritis (MPGN) is a group of autoimmune renal disorders that share certain common histologic features. Until a few years ago, the diagnosis of MPGN was based on ultrastructural findings, specifically the interposition of the mesangium and a double-contoured appearance of the glomerular basement membrane (GBM). MPGN was classified as type I (1), type II (2), and type III (3). Type I was characterized by immune deposits in the mesangium and subendothelial space, while type II, also known as dense deposit disease (DDD), was identified by dense ribbon-like deposits along the basement membranes of the glomeruli and tubules. Type III exhibited subepithelial deposits and subendothelial deposits. However, this classification failed to distinguish between immune complex-mediated from complement-mediated MPGN, which are characterized by overactivation of the alternative pathway of complement (4, 5). In recent years, recognizing the importance of differentiating between these entities for management and treatment, MPGN is considered as a histological “pattern of injury” characterized principally by an increased number of intraglomerular cells and diffuse thickening of the glomerular capillary walls, rather than a specific disease. Thus, a pathophysiological classification based on immunofluorescence findings for immunoglobulin and complement deposition has been proposed (5, 6). According to this new classification, MPGN may be caused by immune complexes, dysregulation of the complement alternative pathway, or, rarely, chronic relapsing endothelial injury in thrombotic microangiopathy without deposits of complement or immunoglobulins. Based on the immunofluorescence characteristics, MPGN can be divided into an immunoglobulin subgroup with or without complement dysregulation, and a complement-dominant subgroup, with absent or minimal immunoglobulin deposits on immunofluorescence. The complement-dominant subgroup may be associated with conditions, such as thrombotic microangiopathy, antiphospholipid antibody syndrome, sickle cell anemia, and polycythemia. Immunoglobulin-dominant MPGN is often associated with autoimmune diseases, chronic infections, or monoclonal gammopathies with or without cryoglobulins. Cases in which the sole or dominant immunoreagent in the renal tissue is C3 or C4 are classified as primary GN and are defined as C3 glomerulopathy or C4 glomerulopathy (7). The morphological phenotypic aspects of C3 glomerulopathy may be either those of DDD, characterized by dense osmiophilic deposits, or those of C3 glomerulonephritis (C3GN), which isolated deposits of C3 with absent or scanty deposits of immunoglobulins (2, 8, 9). Although many reports consider C3 glomerulopathy as a single disease, in this study, we preferred to keep distinct DDD and C3GN.

## Dense deposit disease

DDD is a rare autoimmune kidney disease that primarily affects children and adolescents of both genders. It is characterized by fluid-phase dysregulation of the alternative pathway of complement cascade, either acquired or genetic, leading to low levels of C3 in serum and C3 accumulation in the glomeruli.

### Clinical manifestations

The clinical presentation of DDD can vary. Most cases are characterized by proteinuria, often in a nephrotic range, and hematuria, and half of the cases also present hypertension (10, 11). In rare instances, DDD may mimic renal vasculitis or present with acute kidney failure or crescentic glomerulonephritis (12–14). Serum C3 levels are typically decreased, while serum C4 remains normal. In a few patients, the diagnosis of DDD may be preceded or followed by acquired partial lipodystrophy, also called Barraquer–Simons syndrome, which involves the loss of fat in specific areas of the body, from the face to arms and thorax. The loss of fat may be due to the lysis of adipocytes expressing factor D induced by C3 nephritic factor (C3NeF) (15). Additionally, some patients with DDD may develop drusen, which are extracellular deposits located in the macula of the eye similar to the deposits observed in the kidney (16, 17).

### Pathology

**Figure 1:**
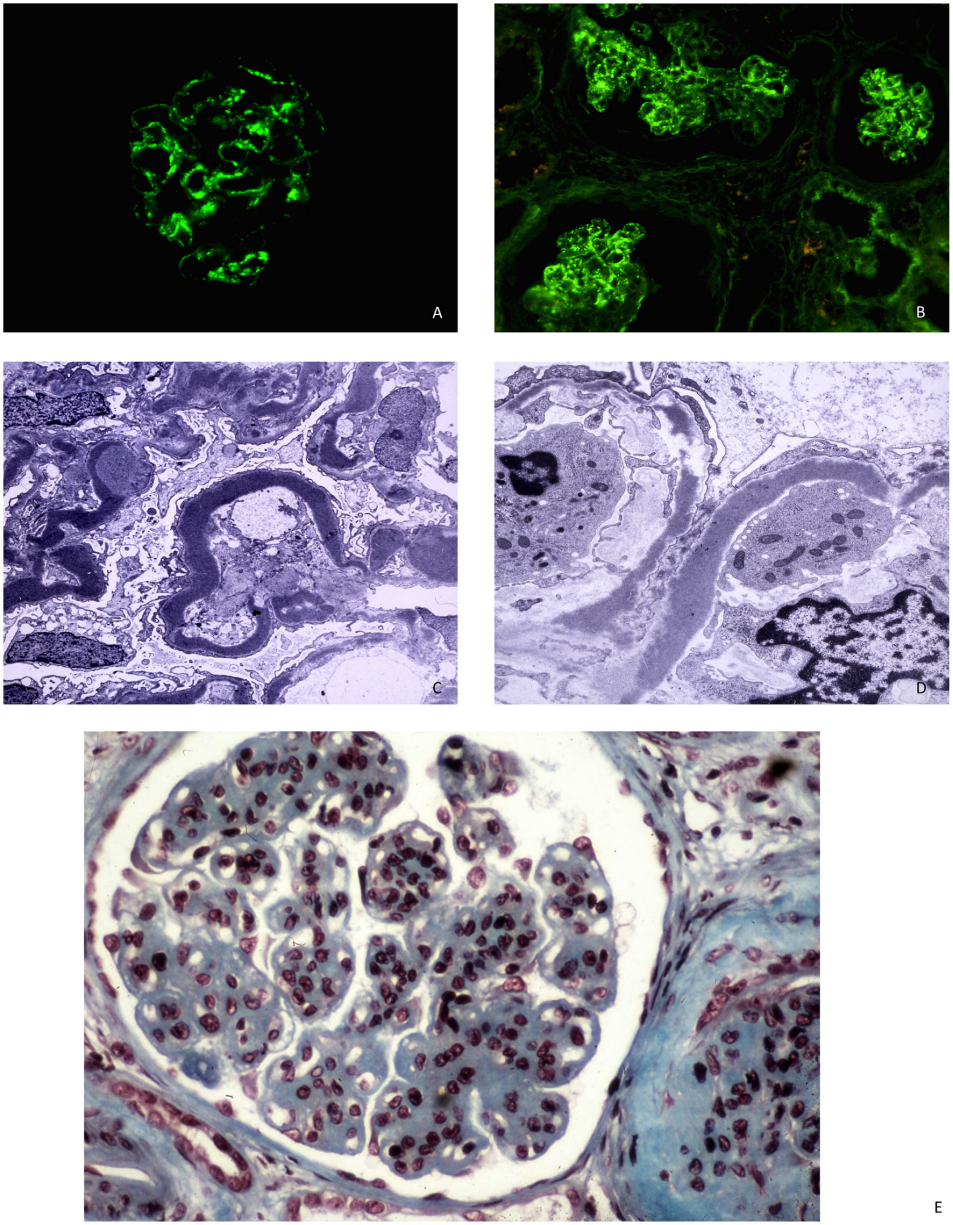
**(A, B)** C3 DDD with mesangial and capillary staining for C3 in immunofluorescence microscopy. **(C, D)** DDD with ribbon-like deposits along the basement membrane lamina densa at electron microscopy. **(E)** The glomerulus shows mesangial hypercellularity and increase in mesangial which gives lobulated appearance and capillary wall thickening. Light microscopy, AFOG's trichrome.

DDD is traditionally classified as MPGN but can exhibit various findings on light microscopy, including mesangial proliferative glomerulonephritis, acute proliferative and exudative glomerulonephritis, crescentic glomerulonephritis, and MPGN. The most common lesion observed under light microscopy is mesangial proliferation with double contours of GBM and cellular interposition. These cellular interpositions lack the eosinophilic appearance typical of immune complex deposits. The GBM segments may stain weakly with Jones silver stain, resulting in a refractile, ribbon-like appearance of the GBM (18). Immunofluorescence staining shows dominant C3 deposition, with little to no deposition of immunoglobulins. C1q and C4d deposition is typically absent or weak. However, the presence of C3 alone has been considered insufficient to define C3 glomerulopathy, and a proposed definition requires C3 dominant and at least two orders of magnitude more intense than any other immune reactant (19). Electron microscopy reveals dense deposition of the basement membrane lamina densa, forming irregular ribbons that transform the GBM into a sausage-like appearance. These deposits contain complement factor C3 as well as components of the alternative pathway and terminal complement complex (20). Deposits may also be found in tubular basement membranes, Bowman's capsule, and mesangial dense globular deposits (Figure 1).

### Complement activation

**Figure 2:**
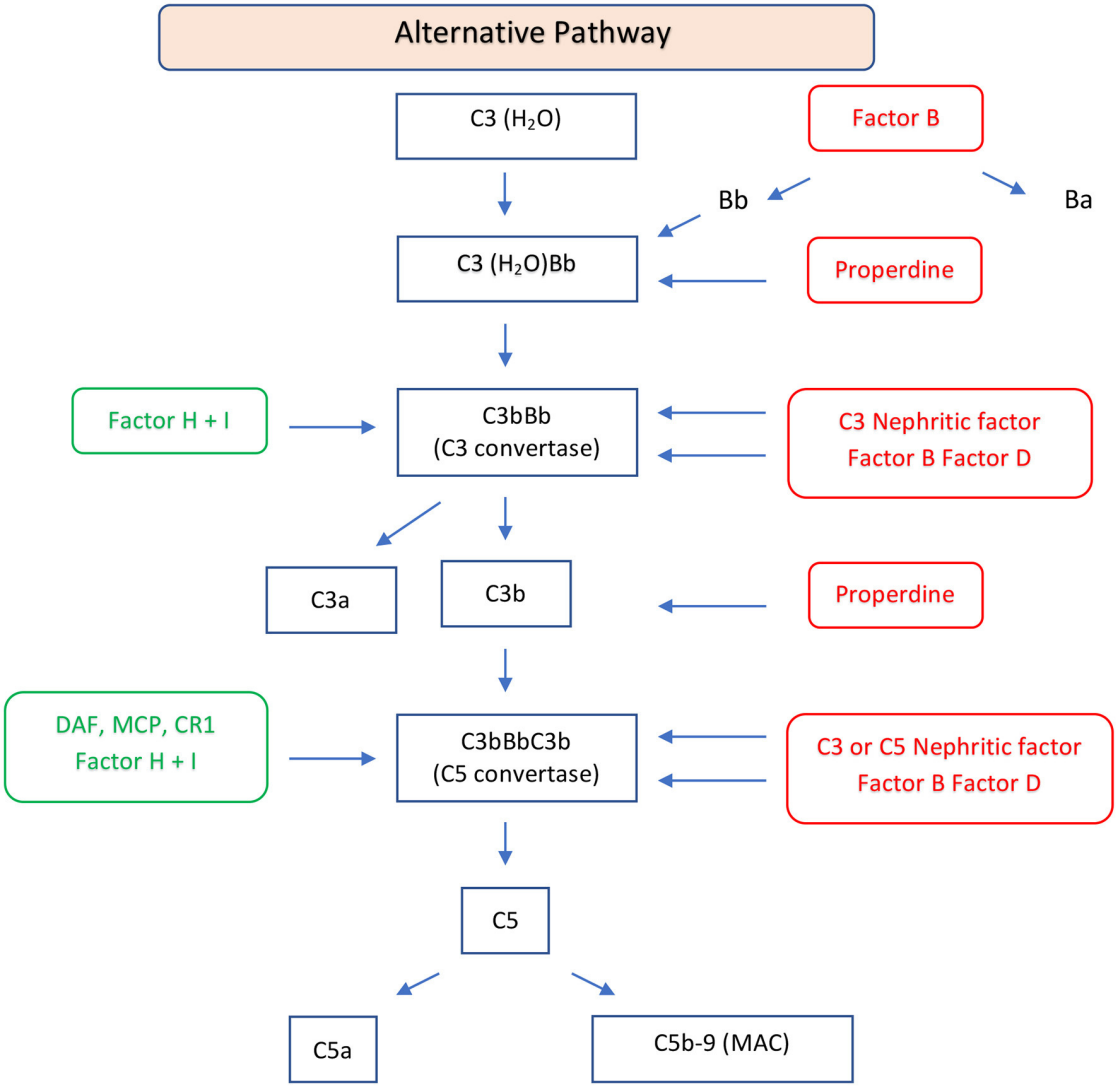
Schematic diagram of the alternative pathway of the complement system. The alternative pathway is constitutively active and is upregulated by binding of C3b to an activating surface. When C3b binds to an activating surface, it triggers a cascade of events, leading the formation of C3 convertase (C3bBb). C3 convertase cleaves C3 in C3a, an anaphylatoxin, and C3b, an opsonin. C3b may form C5 convertase (C3bBbC3b) with generation of C5a, an anaphylatoxin, and C5b, that forms the C5b-9, MAC. This pathway is regulated by regulator factors (in green) to prevent the excessive activation. On the other hand, the promoters or positive regulators factors (in red) play a role in prolonging the activation of the pathway, ensuring a balanced and effective immune response. FB, factor B; FD, factor D; P, properdin; FH, factor H; FI, factor I; DAF, decay-accelerating factor; MCP, membrane cofactor protein; CR1, complement receptor 1; MAC, membrane attack complex.

Complement involvement in DDD is characterized by abnormal activation of C3 and C5 convertase caused by genetic or more frequently acquired factors. C3 convertase can be activated through the classical, lectin, or alternative pathways. In the classical or lectin pathways, C3 convertase (C4b2a) is formed by the cleavage of C4 and C2 mediated by serine proteases. When these two pathways are activated, the C5 convertase (C4b2aC3b) is also formed, resulting in the production of potent anaphylatoxins (C5a), and in C5b which forms a complex with C6 to produce the membrane attack complex C6-C9 (21). In the alternative pathway, the cleavage of C3 leads to the formation of C3a and C3b. The C3b fragment binds a plasma protein called factor B, which is cleaved by factor D to form fragments Ba, and properdin, a glycoprotein, promotes the association of C3b with factor B to form C3Bb, the alternative C3 convertase (Figure 2). Factor H and factor I play a crucial role in regulating the activation of C3 in DDD. Factor H inhibits the formation of the alternative pathway C3 convertases and promotes their dissociation, while factor I degrades factor B, inhibiting alternative pathway C3 convertase formation (22). Mutation of factor H can lead to dysregulation of C3 convertase control, resulting in increased complement activity (23). Autoantibodies against factor H, also known as nephritis factors (NeFs), can stabilize the C3 and C5 convertase, increase their half-life, and may also inhibit factor H, decay accelerating factor, and complement receptor 1 (24–26). C3NeF activity is found in 80% of patients with DDD (27–29), although C3NeFs may also be seen in infections and other autoimmune diseases (30). Properdin can increase the half-life of C3 convertase, facilitate the switch to C5 convertase, and activate the C3 factor of the complement system (31, 32). Moreover, mutations of C3 are associated with dysregulation of C3 convertase (33). In these patients, C3b can act as a substrate for C3 convertase, leading to the formation of C5 convertase. The C5 convertase then cleaves C5 into C5a and C5b. Furthermore, there have been reports of the presence of anti-C3b and anti-factor B IgG antibodies in three patients with DDD (34, 35). There are rare cases of C3-negative DDD characterized by C4d deposition associated with large osmiophilic subendothelial dense deposits (36).

### Differential diagnosis

Complement serology, specifically low serum C3 levels and normal serum C4 levels, is crucial for the diagnosis of DDD. In children, the clinical presentation with pharyngitis, macroscopic hematuria, and hypocomplementemia may resemble post-streptococcal glomerulonephritis, and also, the histological picture of these two diseases is similar. The differential diagnosis may be difficult as the immunoglobulin deposits may be lost with persistent C3 deposits in infection-related diseases. In addition, there are isolated reports in which C3 glomerulopathy was triggered by an infection from streptococci or other pathogens (37–40). With these exceptions, complement levels tend to return to normal with a favorable prognosis in acute glomerulonephritis, while hypocomplementemia is persistent and kidney disease tends to progress in DDD. DDD may also mimic small vessel vasculitis (12), lupus nephritis (41), or hemolytic uremic syndrome (42). To further complicate, distinguishing DDD from C3GN can be challenging. Serum levels of C3 and C4 are similar in both disorders, but capillary wall deposits are observed in C3GN, while DDD exhibits ribbon-shaped dense osmiophilic intramembranous and mesangial deposits. However, the discontinuous nature of ribbons in DDD and sampling errors in biopsies can sometimes lead to misdiagnosis. A cluster analysis has been proposed (see later).

### Outcome and prognosis

DDD is a progressive disease that often may lead to end-stage renal disease (ESRD). Different studies have shown that a significant proportion of patients with DDD progress to ESRD within several years. In an American study of 24 patients, 42% developed ESRD after a mean follow-up of 72 months. Prognostic factors for progression include estimated glomerular filtration rate (eGFR) at diagnosis, tubular atrophy, interstitial fibrosis, and total activity and chronicity scores based on histological findings (43). In a multicenter Spanish study, 43% of 18 patients developed kidney failure after a mean follow-up of 65 months. Baseline eGFR, proteinuria, and immunosuppression therapy were the main determinants of kidney failure. Tubular atrophy, interstitial fibrosis, and the chronicity score were identified as histological predictors (44).

### Treatment

Treatment aims to control blood pressure and reduce proteinuria. According to the KDIGO guidelines, supportive measures are recommended for all patients, while the use of immunosuppressive therapy is reserved for those with moderate-to-severe disease (proteinuria > 1 g/day, hematuria, or decline of kidney function for at least 6 months) (7). Mycophenolate mofetil and glucocorticoids are the preferred initial treatment in these cases. Considering the role of complement dysregulation, targeting the normalization of the alternative complement pathways has also been proposed (45). Therapies such as plasmapheresis, sulodexide, rituximab, and eculizumab have been used in some cases, but their efficacy varies. Eculizumab, a chimeric humanized monoclonal antibody against the C5 component of complement, inhibits the ability of C5 convertase to cleave C5 to C5a and C5b, thus preventing the formation of the membrane attack complex C5b-C9. Eculizumab seemed to be effective in some patients, but in some studies, the rate of remission was inferior to steroids and mycophenolate mofetil (46–51). In addition, patients may relapse when eculizumab is discontinued and relapses do not respond to further administration of eculizumab (52). Trials exploring the efficacy of new drugs inhibiting complement activation, such as iptacopan, an inhibitor of factor B (53), danicopan, an inhibitor of factor D (54), pegcetacoplan, an inhibitor of C3 and C3b (55), and avacopan, an inhibitor of C5a, are underway [ClinicalTrials.gov].

### Recurrence after kidney transplantation

Recurrence of DDD in kidney transplant recipients is common, and (10, 15) few data are available about the outcome and the treatment of recurrence of DDD. One of the largest groups of kidney-transplanted patients with C3 glomerulopathy included seven cases of DDD. Recurrence occurred in six patients and caused graft loss in four of them (56). A systematic review and meta-analysis assessed the efficacy of different therapeutical approaches for the recurrence after kidney transplant in 122 patients with C3 glomerulopathy. Among them, 49 patients with DDD were included. Allograft loss was recorded in 53% of patients who were given eculizumab, in none of the two patients treated with plasma exchange within 6 months after recurrence, and in all three patients treated with rituximab. Of note, 53% of patients with DDD who did not receive treatment for the recurrence lost the graft (57).

## C3 glomerulonephritis

According to the current classification, C3GN is a proliferative GN, characterized by the presence of C3 staining on immunofluorescence and mesangial or subendothelial deposits on electron microscopy.

### Clinical manifestations

The clinical presentation is variable, similar to that observed in DDD. Patients with C3GN tend to be older and have less renal impairment at presentation compared to DDD. C3NeF is found in ~45% of patients with C3GN (27). However, hematuria, severe proteinuria, hypertension, and low C3 complement levels are common in both diseases (58). Acquired partial lipodystrophy and Bruch's membrane involvement are specific to DDD and not seen in C3GN. Differential diagnosis can be challenging in older patients with C3GN who may have a monoclonal gammopathy, which requires further diagnostic tests, such as serum protein electrophoresis, serum and urine immunofixation, and research for serum free light chains levels to evaluate the presence of an underlying pathological plasma cell or B-cell clone (59, 60). In addition, post-infectious glomerulonephritis can mimic C3GN, but hypocomplementemia and proteinuria tend to normalize and C4d staining is positive in post-infectious glomerulonephritis (61–63).

### Pathology

Using light microscopy, C3GN typically shows a classical pattern of MPGN with mesangial and endocapillary proliferation, double-contour formation of the capillary wall, and lobular accentuation of the capillary tufts. However, some cases may initially present endocapillary proliferative GN without mesangial change. Crescents are more frequent in DDD, while chronicity markers such as glomerular sclerosis, arteriolar sclerosis, and interstitial fibrosis are more common in C3GN (11). Immunofluorescence shows the presence of C3 deposits without other immune deposits or faint deposits of immunoglobulins. The intensity of C3 deposits should be at least two orders of magnitude more intense than any other immune reactant (19). Using electron microscopy, hump-like and clustered deposits in the mesangium or in the subendothelial and/or subepithelial spaces resulting in irregular thickening of the glomerular basement membrane were found (64). Sometimes, however, it can be difficult to distinguish an immunoglobulin-associated MPGN from a C3GN as both disorders can exhibit similar abnormalities of the alternative pathway of complement.

### Complement activation

Dysregulation of the alternative pathway of complement can occur due to genetic mutations of factor H or the presence of autoantibodies. The C3NeFs are present in 40–50% of patients with C3GN (26, 65, 66). Compared with DDD where C3NeFs may be properdin independent, in C3GN most C3NeFs are properdin dependent (67). Nephritic factors are persistently present and are associated with complement activation, but their serum levels may be variable over the course of DDD or C3GN (68). High levels of C3NeFs are associated with active disease, whereas their decline, in response to plasma exchanges and/or immunosuppressive therapy, is associated with disease remission (69). It should be noted that autoantibodies against factor H are usually associated with homozygous deletions in the genes encoding the factor H and can be found also in children with atypical hemolytic uremic syndrome (28, 70, 71). As reported above, both C3 GN and Ig-associated MPGN can exhibit directed against factor B and/or C3b leading to alternative pathway activation, suggesting similar pathogenetic mechanisms (72).

### Differential diagnosis

Persistent hypocomplementemia with low serum levels of C3 and normal levels of C4 is characteristic of C3GN. Overlap between C3GN and thrombotic microangiopathy is extremely rare and associated with poor kidney outcomes (58, 73). Monoclonal gammopathy is often present, and the phenotype may show either the characteristics of a C3GN or those of hemolytic uremic syndrome (74, 75). The discovery of C3GN in an older adult should always initiate a search for a monoclonal gammopathy. In positive cases, it is important to evaluate whether the immunoglobulin is secreted by a premalignant or malignant clone. Kidney biopsy is essential to better define the characteristics of monoclonal gammopathies of renal significance. Light microscopy may show a membranoproliferative injury pattern with minimal mesangial hypercellularity and rare subepithelial “hump-like” deposits. Immunofluorescence may be positive for C3 in a granular mesangial distribution, but immunoglobulin deposition may be present or absent. The electron microscopy may reveal whether the organized deposits are fibrillar or microtubular. A spectrum of diseases may be present in patients with a suspected monoclonal gammopathy of renal significance, including thrombotic microangiopathy, cryoglobulinemia, amyloidosis, immunotactoid glomerulopathy, and fibrillary glomerulonephritis (76, 77). A correct diagnosis is of capital importance to establish the prognosis and management of these patients, so it is essential to investigate the presence of monoclonal gammopathy in older adults with C3GN. Cluster analysis has been proposed to differentiate DDD from C3GN and immune complex GN (ICGN), based on histologic, genetic, clinical, and complement phenotypes. Iatropoulos et al. arranged 173 patients with C3G or ICGN into four different pathogenetic patterns (clusters). Patterns 1, 2, and 3 exhibited fluid-phase complement activation, low C3 levels, and a high prevalence of genetic and acquired alternate pathway abnormalities. Pattern 4 had solid-phase complement activations and usually normal C3 levels. Patterns 1 and 2 had evidence of concomitant classical pathway activation, but patients in cluster 2 had additional activation of the classic pathway and the highest prevalence of nephrotic syndrome at disease onset. Pattern 3 was associated with prominent activation of C3 convertase and features of DDD characterized by electron microscopy. This type of analysis can be applied in an algorithmic fashion to improve risk assessment and clarify disease pathogenesis (78).

### Outcome and prognosis

A diagnosis of C3GN indicates a higher risk of progressing to ESRD. However, chronic renal failure progresses slower in adults with C3GN than in younger patients with DDD. In a study, no patient with DDD was still alive with kidney functioning at 10 years vs. 82% of patients with C3 GN. The eGFR at presentation is strongly correlated with kidney survival (79). Other series reported that persistent hypertension and proteinuria are associated with a poor outcome (80). A multicenter French multivariate analysis on 165 patients with C3GN reported that low C3/normal C5b-9 levels and normal C3/high C5b-C9 levels were predictors of worse kidney prognosis, with the relative risk 3.7- and 8-times higher, respectively (81). In addition, the diagnosis of monoclonal gammopathy is crucial for the outcome and treatment of patients with C3GN (82). In the absence of specific treatment, these patients with C3GN and monoclonal gammopathy progress to ESRD (83).

### Treatment

Managing C3GN focuses on preserving kidney function. The use of angiotensin-converting enzyme inhibitors or angiotensin receptor blockers may be beneficial for renal survival (84). In a multicenter Spanish study, combination therapy with glucocorticoids and mycophenolate mofetil (MMF) has shown promising results in achieving remission of proteinuria. These data were demonstrated in a Spanish study and confirmed in another one from Turkey and the United States (85, 86). In this last study, patients treated with MMF and prednisone had higher response rates than those treated with glucocorticoids alone or combined with calcineurin inhibitors, rituximab, or alkylating agents plus corticosteroids. Eculizumab may be effective in rapidly progressive forms of C3GN but has limited benefits in other cases (87), and in a large Spanish review, eculizumab obtained less remission than glucocorticoids and MMF (88). A phase 2 study was unable to demonstrate a significant inhibition of the alternative pathway in C3GN with danicopan, an inhibitor of factor D (54). Ongoing clinical trials are investigating the use of new drugs targeting complement activation in C3GN. Treatment of patients with monoclonal gammopathy, without pathologic clone, is not well-established. Daratumumab, a human monoclonal anti-CD38 antibody, used in the treatment of multiple myeloma appears to be a possible treatment option for these patients. In an open-label phase 2 clinical trial, it is associated with improvement of proteinuria and stabilization of kidney function (89).

### Recurrence after kidney transplantation

Recurrence of C3GN after kidney transplantation is common, although there are limited data available regarding the outcomes and treatment of these patients (90). Recurrent C3GN after kidney transplantation has been associated with graft loss, especially in cases with underlying monoclonal gammopathy. Treatment options vary, including eculizumab, plasma exchange, and rituximab, but the efficacy of these interventions requires further evaluation. Regunathan-Shenk et al. (56) described recurrence in 10 of 12 C3GN-transplanted patients in the median of 76 months after transplant and three grafts were lost for recurrence. In a previously reported systematic review about the treatment of recurrent C3 glomerulopathy after kidney transplant, of 73 patients with C3GN, allograft loss occurred in 22% of recurrent patients treated with eculizumab, 56% of those treated with plasma exchange, 70% of patients receiving rituximab, and 32% of recurrent patients who did not receive therapy due to stable allograft function (57). A high rate of recurrence (67%) has been reported in a study of 21 kidney transplant recipients with C3GN after a follow-up of up to 13 years. The recurrence rate at 1 year of follow-up was 30% with a median time of 32 months. Graft failure was ~50% at 10 years in the 14 patients with a recurrence (all but 3 of which had received living donor transplants). The recurrence rate was particularly high in those patients with an underlying monoclonal gammopathy. Rituximab and stem cell transplantation seemed to be of benefit in patients with monoclonal gammopathy (82).

## Conclusion

C3 glomerulopathy is an uncommon progressive disease that can lead to ESRD. C3GN primarily affects adults, whereas DDD is more commonly observed in pediatric patients. The prognosis is generally worse in children with DDD compared to adults with C3Gn as the disease progresses more rapidly in pediatric cases. Although DDD and C3GN share similarities in terms of clinical presentation and histological features, there are distinct pathological characteristics and extra-renal features that help differentiate between the two conditions. In C3GN, there is typically more severe arteriolar and glomerular sclerosis and interstitial fibrosis. On the other hand, DDD is characterized by the presence of ribbon-like deposits on electron microscopy, which is essential for the diagnosis. These differences in histological findings can be significant in the context of prognosis and treatment outcomes, warranting separate descriptions for these uncommon entities (Table 1).

**Table 1:** Main clinical and histologic features of DDD and C3GN.

	**Dense deposit disease**	**C3 Glomerulonephritis**
Epidemiology	Primarily children and young patients M = F	Primarily adult patients M = F
Clinical presentation	Proteinuria (often in nephrotic range), glomerular hematuria and/or hypertension Reduced GFR Persistent low C3 with normal C4.C3NeF in 80% of patients. Often, associated with acquired partial lipodystrophy and drusen.	Proteinuria, glomerular hematuria and/or hypertension Reduced GFR Persistent low C3, normal C4.C3NeF in 40–50% of patients Frequently monoclonal gammopathy is present (exclude hematological disease)
Renal Pathology	LM: Mesangial proliferative GN with double contours of GBM and cellular interposition Ribbon-shaped dense osmiophilic intramembranous and mesangial deposits Crescents more frequent than in C3GN IF: Dominant C3 deposition (at least two orders of magnitude more intense than any other immune reactant) EM: Linear-appearing, highly electron-dense deposits in the GBM, tubular basement membranes, Bowman's capsule and mesangium	LM: Mesangial proliferative GN with mesangial and endocapillary proliferation, double contours of the capillary wall Lobular accentuations of the capillary tuft Chronic lesions more frequent than in DDD. IF: Dominant C3 deposition (at least two orders of magnitude more intense than any other immune reactant) EM: hump-like and clustered deposits in the mesangium or in the subendothelial and/or subepithelial spaces. Irregular thickening of the GBM
Outcome and Prognosis	Common ESRD. Most patients in ESRD within 10 years	Common ESRD, but slower progression than DDD
Recurrence after transplant	High rate of recurrence after transplant	High rate of recurrence after transplant, especially in pts with monoclonal gammopathy

GFR, glomerular filtration rate; LM, light microscopy; GBM, glomerular basement membrane; IF, immunofluorescence; EM, electron microscopy; ESRD, end stage renal disease; C3NeF, C3 nephritic factor.

In summary, the age of onset, clinical features, and histological findings of DDD and C3GN vary, suggesting the need for differentiation between the two conditions.

## Author contributions

CP: Conceptualization, Writing – original draft. MC: Writing – review & editing. GM: Supervision, Writing – review & editing.
